# Improved lyophilization conditions for long-term storage of bacteriophages

**DOI:** 10.1038/s41598-019-51742-4

**Published:** 2019-10-23

**Authors:** Prasanth Manohar, Nachimuthu Ramesh

**Affiliations:** Antibiotic Resistance and Phage Therapy Laboratory, School of Bio Sciences and Technology, Vellore Institute of Technology (VIT), Vellore, 632014 Tamil Nadu India

**Keywords:** Bacteriophages, Phage biology

## Abstract

Phage therapy is one of the promising alternatives to combat the increasing problem of antibiotic resistance. Lyophilization is used for the preparation of pharmaceutical products to improve their stability in long-term storage. The aim of this study was to improve the stability of lyophilized bacteriophages using different excipients. Three lytic bacteriophages *Escherichia* phage ECP311, *Klebsiella* phage KPP235 and *Enterobacter* phage ELP140 were subjected to lyophilization using six different excipients: glucose, sucrose, gelatin, mannitol, polyethylene glycol and sorbitol. The lyophilized phages were stored at 4 °C and 37 °C and rehydrated using biological saline to test their viability at 5 months interval up to 20 months. The results showed that the use of sucrose, gelatin and their combination was beneficial in maintaining the viability of phages post-lyophilization. When lyophilized phages were stored at 4 °C, their viability was maintained up to 20 months, but at 37 °C there was a reduction in activity after 10 months. This is one of the few studies to report the lyophilization of phage cocktails to have viability for up to 10 months. Our study identified promising lyophilization excipients to effectively lyophilize bacteriophages for pharmaceutical applications and long-term storage.

## Introduction

Developing resistance among bacteria towards all the available antibiotics makes the clinical treatment of infections more serious and difficult^1^. Alternative therapy is required to control the dissemination of antibiotic-resistant bacterial infections. Bacteriophage use has potential as alternative therapy to antibiotic treatment, hence the renewed scientific interest in its clinical applications^2–7^. There is a wide range of animal studies and clinical trials that could prove and support phage therapy as a good alternative to antibiotics^4–8^. However, despite evidence of therapeutic efficacy, potential pharmaceutical formulations for long-term storage and treatment are lacking^2,9,10^. Bacteriophages are viruses made of proteins and nucleic acids, which can lose their viability during long-term storage especially in suspension^3^. Phages are primarily protein particles (protein capsids); therefore, phage formulations can reasonably have similarities to other protein formulations. Majority of the proteins are more stable in the dry state than in solutions^11^. Lyophilization of proteins can provide more stability through reducing the molecular mobility, hydrolysis and contamination but physical and chemical instabilities are not clear. During protein lyophilization it is important to consider their conformation (protein structure), and chemical and physical stability as well as their ability to completely dissolve upon reconstitution. Many therapeutically important proteins and vaccines are lyophilized to obtain therapeutic pharmaceutical formulations^3,7^. Phages can undergo physical stresses in aqueous solution (suspensions) such as pH/temperature changes, agitation and exposure to denaturants, which can lead to aggregation and phage loss. To maintain stability during long-term storage of phages, preparation of lyophilization powders is one of the effective mechanisms that need more exploration. Earlier studies showed that the lyophilized phages are stable at −80 °C for up to 10 years, though there was a decline in activity of 1 log_10_/year^12^.

Additionally, excipients used for lyophilization are one of the important criteria for effective phage lyophilization and also for their viability^13,14^. Earlier studies showed the use of skimmed milk, gelatin, peptone, sodium glutamate, polyethylene glycol, glycerol and other sugars (mannitol, sucrose, and trehalose) as effective excipients for phage lyophilization^3^. Sugars are considered as good excipients for phage lyophilization because they can stabilize phages during lyophilization process and also help in shelf storage of phage products^15–18^. A recent study by Zhang *et al*., showed the use of trehalose and PEG as potential excipient for phage lyophilization^14^. The majority of the studies on phage lyophilization showed less than 2 log reduction in phage titer when stored at 4 °C but it is important to study the viability of phages at ambient temperatures (35–37 °C) for long-term storage. It should also be noted that the order *Caudovirales* (tailed phages) has three different families (*Myoviridae*, *Siphoviridae* and *Podoviridae*), based on different morphology. When phages are lyophilized for therapeutic purpose, the morphology of these phages should be retained in order to achieve the viability. Therefore, the choice of excipients used for lyophilization of different families of tailed phages should be taken into consideration.

In this study, we examined the lyophilization technique using six different excipients for preparing viable phage powders. The chosen excipients were mostly sugars because sugars can act as good cryoprotectants which have an ability to undergo vitrification during freezing, eventually forming glass matrix that can prevent phages from aggregation^19^. The six excipients chosen for this study were based on the previous reports^2,3,7^ and the combination of these excipients was tested for the first time in this study. We also evaluated the viability of phages for long-term storage up to 20 months at 4 °C and 37 °C, and lytic activity of these phages was determined before and after lyophilization. Additionally, a phage cocktail was lyophilized and the viability was evaluated up to 20 months at 4 °C and 37 °C.

## Results and Discussion

### Viability of phages after lyophilization

**Figure 1:**
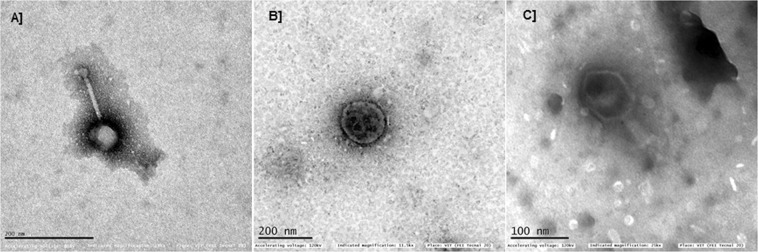
Transmission Electron Microscopic (TEM) images of (**A**) *Escherichia* phage- ECP311, (**B**) *Klebsiella* phage- KPP235, (**C**) *Enterobacter* phage- ELP140.

**Figure 2:**
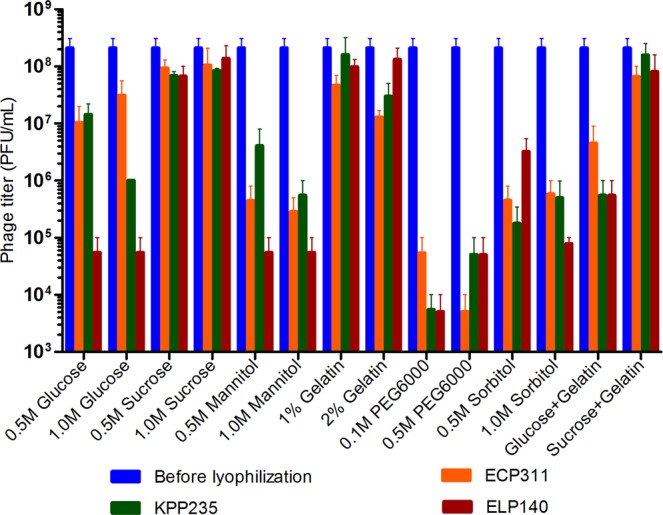
Stability of *Escherichia* phage- ECP311, *Klebsiella* phage- KPP235, *Enterobacter* phage- ELP140 in different excipients after lyophilization. The results are the mean values obtained from two independent replicates. The error bars represent the standard deviation.

In this study, three *Caudovirales* phages with different morphology (Fig. 1) were lyophilized using the two-step process; primary and secondary drying that eventually allows the formation of phage powder, and phage activity (plaque assay) was performed using lyophilized phage powder. In the initial stability study, the phage activity was tested using lyophilized phages and compared to phages in suspension, and the loss in phage viability during lyophilization was noted (Figs 2, S1). For the three tested phages, ECP311 (MG972768), KPP235 (MG983840) and ELP140 (MG999954), sucrose (0.5 M and 1.0 M), gelatin (1% and 2%) and the combination sucrose plus gelatin (0.5 M and 1%) were found to be beneficial in restoring the phage activity post-lyophilization (<1 log reduction in phage activity was observed during lyophilization process) (Fig. 2). However, two previous studies showed contrasting results; a study by Puapermpoonsiri *et al*., stated that the higher concentrations of sucrose lead to decrease in phage titer^3^ and another study by Merabishvili *et al*., showed that phage activity was retained even at higher concentrations of sucrose^2^. These distinct stabilities may be due to different concentrations of stabilizers used during lyophilization and it is also believed that different families of phages having varying morphologies have varying stability and tailed phages are known to be most stable during long-term storage. In this study, sucrose at concentrations, 0.5 M and 1.0 M, was found to be beneficial (<1 log reduction) for all the three phage types and similar results were observed by Merabishvili *et al*., for *Staphylococcus aureus* phage ISP^2^. Sugars, mostly disaccharides, are found to be good excipients for phage lyophilization^2^, and in this study sucrose at both lower (0.5 M) and higher (1.0 M) concentrations was found to be beneficial for phage lyophilization process. Disaccharides such as sucrose are found to have more structural flexibility than monosaccharides, which can support the proteins to maintain their native conformation^20^. Though earlier studies showed the use of trehalose as a good excipient for phage lyophilization, the use of trehalose was not tested in this study because trehalose was not approved to use in pharmaceutical products either as oral or as inhalation powders^21^. Sucrose can act as an excellent excipient for phage lyophilization because (a) they are pharmaceutically acceptable, (b) their exclusion from the proteins during lyophilization^22^, (c) thermodynamically favoring the folded form eventually protecting the phage structure^23^ and (d) they form hydrogen bonding with polar groups on the protein surface^24^. The stability offered by sugars during lyophilization can be improved by using bulk concentrations^3^. The purity of phages may affect the stability because precipitated phages are free of endotoxins (bacterial impurities) that allow easy crystallization of phage proteinaceous particles.

The other excipients such as glucose, mannitol and sorbitol were found to yield reduced titers (Fig. 2). As known glucose, sorbitol and mannitol can be crystallized quickly that can result in destabilization of phages, though earlier study reported the use of mannitol as a good excipient when combined with other sugars^2^. Polyethylene glycol (PEG) was also found to be detrimental for phage lyophilization (Fig. 2). PEG is a polyhydric alcohol that caused greater than 4 log reduction in phage activity post-lyophilization though PEG was considered to be an effective cryoprotectant for other proteins^2^. Usually PEG is found to have hydrophobic interactions with non-polar protein residues and they can precipitate phage particles, therefore, PEG alone is not considered as a very good excipient for phage lyophilization^25^. Earlier studies also showed that PEG can be detrimental during phage lyophilization process and PEG can act as a good excipient when mixed with sugars such as sucrose or mannitol^2,3^.

Gelatin was also found to be a good excipient for all the three phage types (accepting <2 log reduction) studied, and another study^3^ showed that gelatin had better stabilization properties for *Staphylococcus* phage (*Siphoviridae*) and *Pseudomonas* phage (*Myoviridae*). The role of gelatin as an excipient during lyophilization was not clear but we believe that gelatin can form polymers which support to maintain phage morphology during lyophilization. The combination of sucrose (disaccharide) plus gelatin (polymer) at 0.5 M and 1% respectively were tested for the first time in this study, and it was proved to be beneficial (<1 log reduction). Here, the use of disaccharide, sucrose, is believed to stabilize phages during lyophilization by forming a hydrogen bond to the protein surface, and polymers such as gelatin can reduce water retention, thereby, stabilize the effect of sucrose. Polymers can protect proteins and can stabilize the multi-subunit protein structures through preferential hydration of protein from water during lyophilization process^26^. Though earlier studies showed the use of polymer such as PEG as a good excipient^2,3^, this study showed the use of gelatin (alone and in combination) to have a more improved activity as an excipient. This study reports the use of gelatin alone (resuspended in biological saline) as an excipient at concentrations of 1% and 2% that could stabilize the phage titer during the lyophilization process. Earlier, a study by Puapermpoonsiri *et al*., reported the use of SM buffer with gelatin as a good excipient for phage lyophilization^3^ and another study by Shapira and Kohn reported the use of gelatin as an excipient for T4 phage lyophilization^27^.

Lyophilization is a two-step process: in a primary drying step the samples are frozen to −30 °C for 12 hours resulting in the sublimation of phage particles and secondary drying step results in the desorption of remaining water molecules (from −30 °C to 25 °C for 10 hours), leaving the dry lyophilized powder. Residual moisture content was found to be one of the important parameters to obtain a stable lyophilization product (final product). From previous studies, it was observed that the lytic activity of lyophilized bacteriophage depends on the lyophilization process and there was a correlation observed between moisture content in lyophilized powder and lytic activity of phages^3^. Taking this into account, the moisture content in the lyophilized powder was reduced by extending the primary drying step (up to 12 hours), resulting in sublimation of bulk water and also by elevating the temperature (up to 25 °C) during secondary step, to remove water molecules adsorbed to the product surface^28^. To be noted that the moisture content was not studied separately in this study.

### Rehydration and viability of lyophilized phages for long-term storage

**Figure 3:**
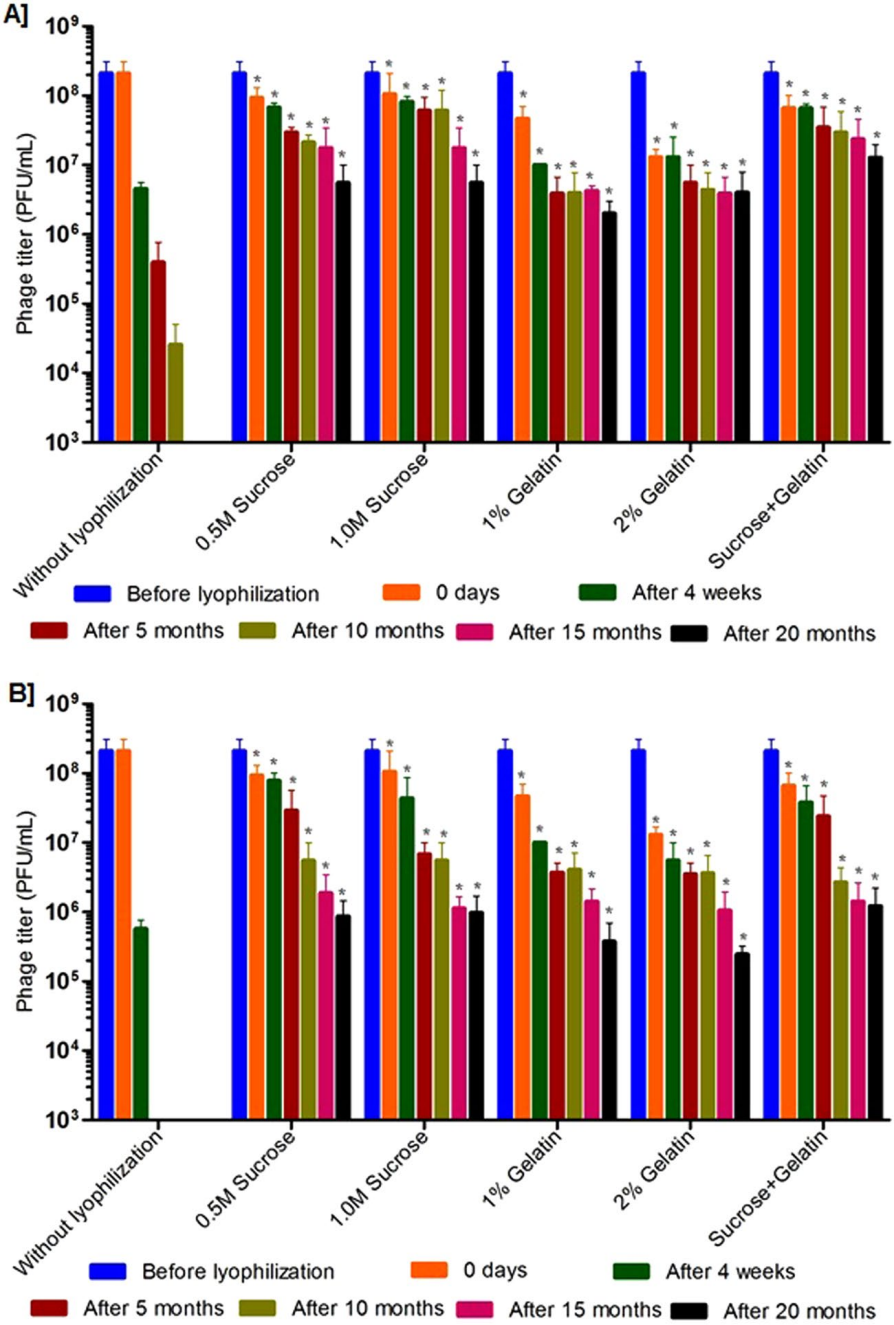
Stability of *Escherichia* phage- ECP311 in two different excipients and their combinations after lyophilization and storage at, (**A**) at 4 °C, (**B**) at 37 °C. The results are the mean values obtained from two independent replicates. The error bars represent the standard deviation. *Represents the results obtained for lyophilized phage.

**Figure 4:**
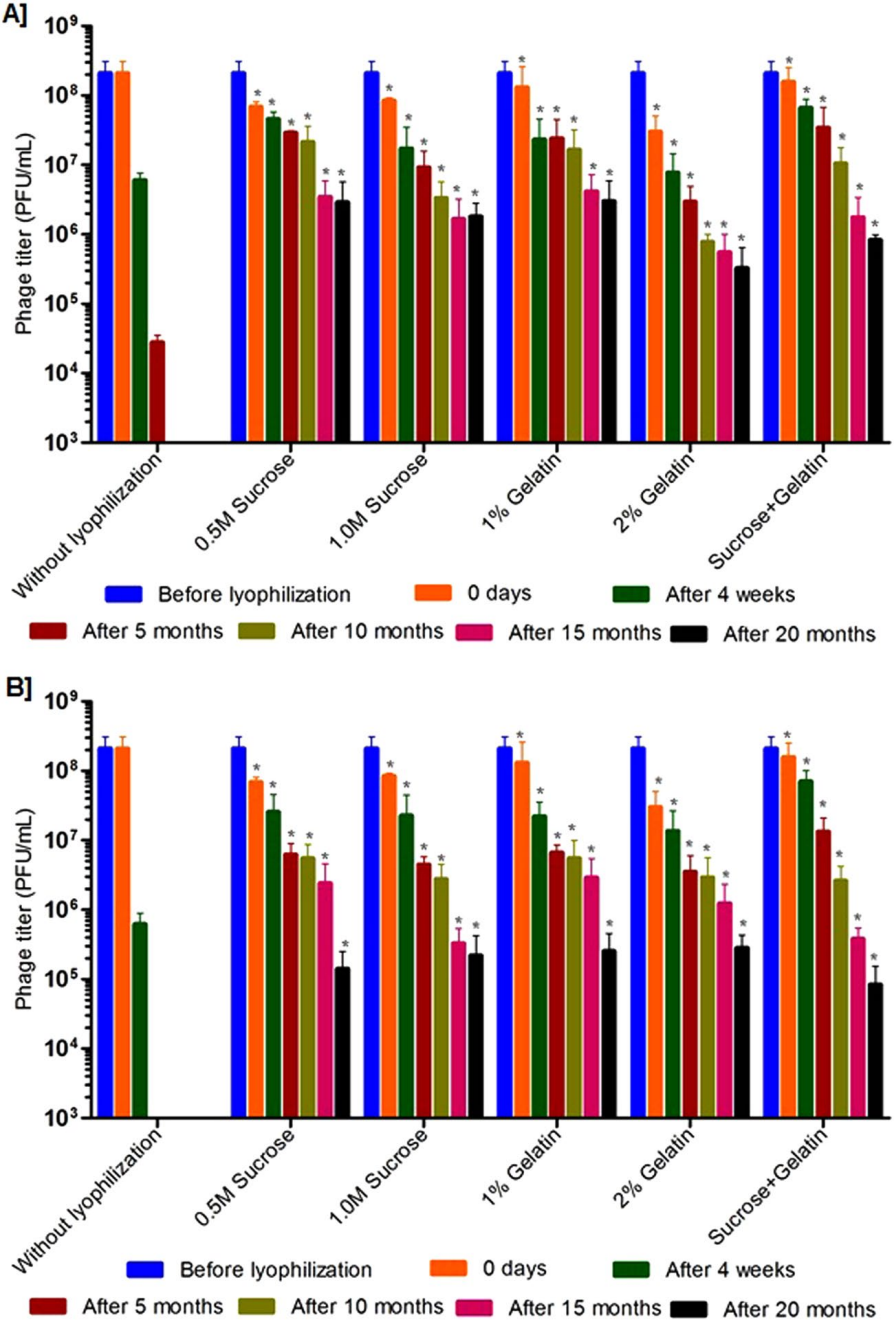
Stability of *Klebsiella* phage- KPP235 in two different excipients and their combinations after lyophilization and storage at, (**A**) at 4 °C, (**B**) at 37 °C. The results are the mean values obtained from two independent replicates. The error bars represent the standard deviation. *Represents the results obtained for lyophilized phage.

**Figure 5:**
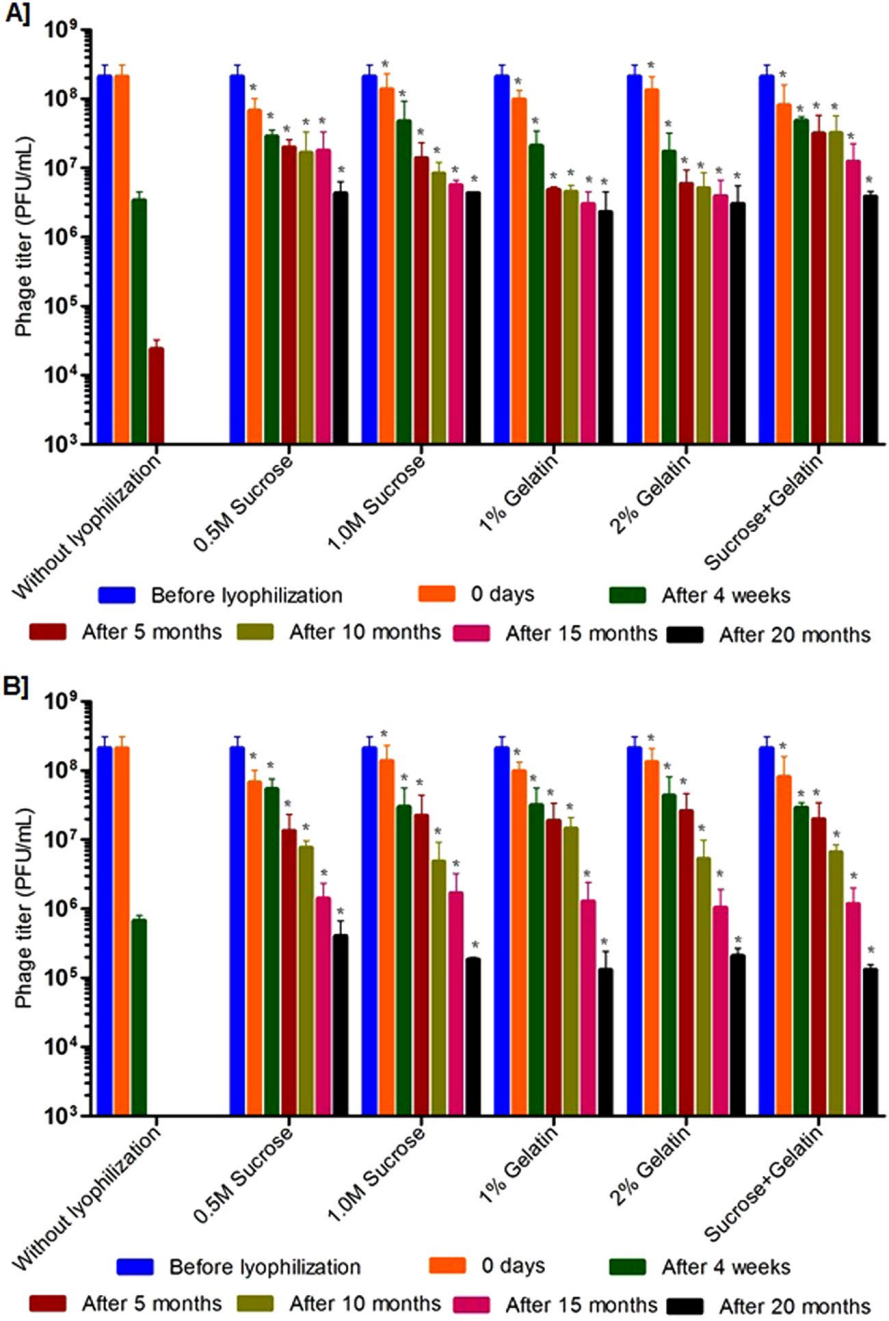
Stability of *Enterobacter* phage- ELP140 in two different excipients and their combinations after lyophilization and storage at, (**A**) at 4 °C, (**B**) at 37 °C. The results are the mean values obtained from two independent replicates. The error bars represent the standard deviation. *Represents the results obtained for lyophilized phage.

Further, long-term stability studies were performed using the phages (ECP311, KPP235 and ELP140) that were lyophilized using sucrose, gelatin and the combination of sucrose plus gelatin. The lyophilized phages were stored at 4 °C and 37 °C and their viability was tested at every 5 months interval up to 20 months, and rehydration was performed using biological saline. In the case of ECP311, the lyophilized phages were viable up to 20 months (with <2 log reduction in activity) when stored at 4 °C (Figs 3, S1). But at 37 °C, the ECP311 was found to lose their activity after 10 months of storage. Similarly, KPP235 and ELP140 had minimal activity when stored at 37 °C for 20 months (Figs 4 and 5, S1) but at 4 °C phages were found to be viable up to 20 months. The negligible loss in phage titer after long-term storage of lyophilized phages showed that sucrose, gelatin and sucrose plus gelatin are beneficial for phage lyophilization. An earlier study by Puapermpoonsiri *et al*., showed the beneficial effect of sucrose in SM buffer to have viable phage titer post-lyophilization^3^, and in our study, the phages that are lyophilized using sucrose as an excipient were found to be viable up to 20 months at 4 °C. Additionally, in this study, the phages were found to be maintaining the high titer up to 10 months when stored at 37 °C which is very important for phage therapy because there are controversies about the stability of phages during long-term storage at ambient conditions i.e. 37 °C^7^. Another study by Merabishvili *et al*., showed that LB broth has a strong influence in maintaining phage viability^2^.

**Figure 6:**
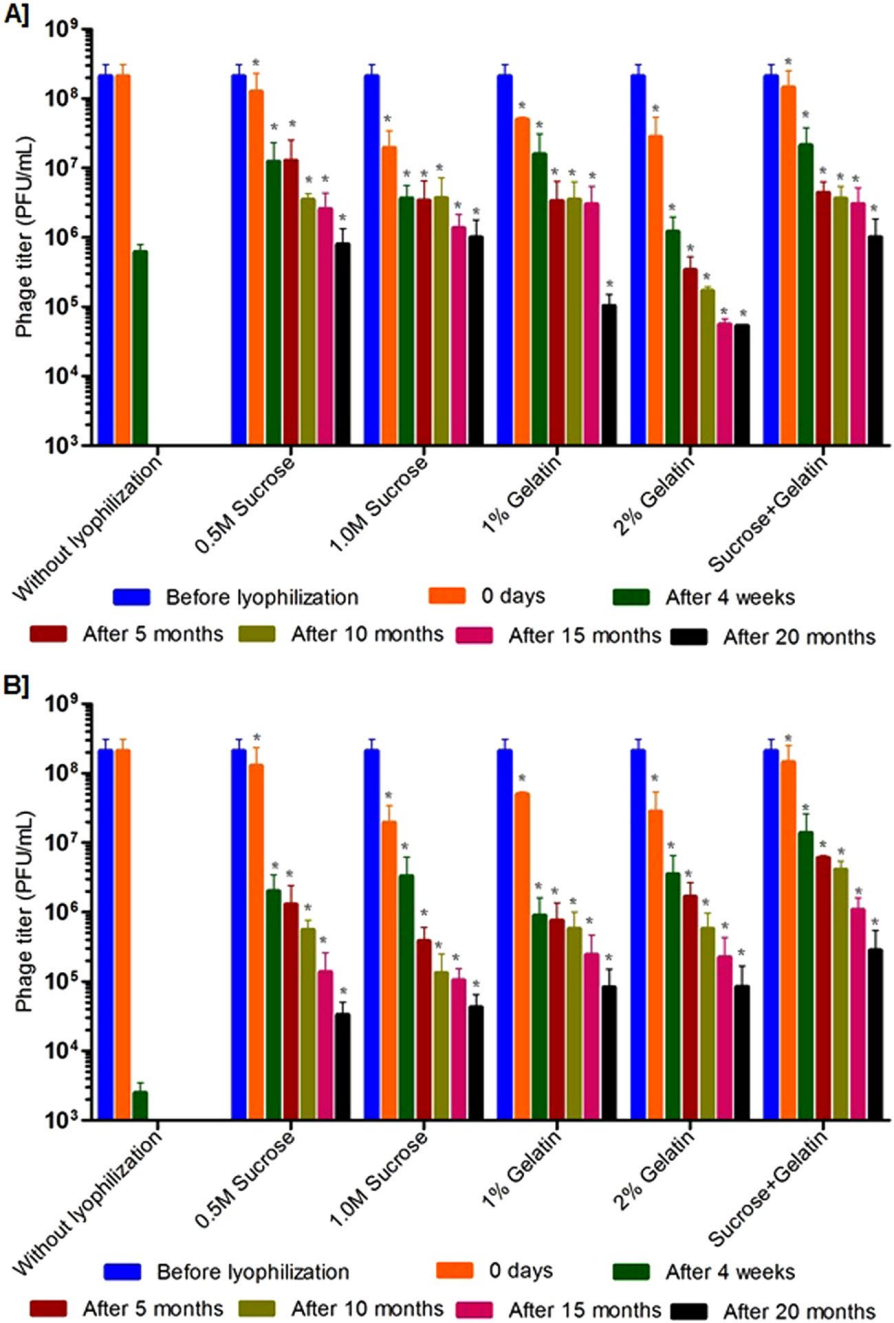
Stability of lyophilized phage cocktail using two different excipients and their combinations and storage at, (**A**) at 4 °C and (**B**) 37 °C. The phage cocktail contains *Escherichia* phage- ECP311, *Klebsiella* phage- KPP235, *Enterobacter* phage- ELP140. The results are the mean values obtained from two independent replicates. The error bars represent the standard deviation. *Represents the results obtained for lyophilized phage.

Rehydration is one of the important processes that should have more attention in phage lyophilization studies. In this study, the lyophilized phages were carefully rehydrated (before testing) using biological saline and phage activity was evaluated. A study by Cox *et al*., explained the importance of phage rehydration post-lyophilization, the study detailed that rapid rehydration may cause detachment of head and tail particles leading to reduced phage viability^29^. A study by Cox *et al*., concluded that slow rehydration (gradual addition of diluent) with the controlled humid environment can protect the viability of phages for long-term storage^29^. As this study deals only with the slow rehydration process, we could not explain the difference in phage viability during slow and rapid rehydration. But slow rehydration and storage at 4 °C and 37 °C did not make any difference in this study because phages were found to be viable at both the conditions (Figs 3, 4, 5 and 6).

This study showed the use of sucrose, gelatin and sucrose plus gelatin as a good excipient during phage lyophilization with <1 log reduction in activity post-lyophilization. But sucrose should be favored as an excipient for phages that are to be used for therapeutic purpose and gelatin (also the combination of gelatin) can be chosen for long-term storage because gelatin is of animal origin hence there is a potential risk of contamination and transmitting animal diseases, therefore, cannot be considered as a good therapeutic agent^2^. After long-term storage of lyophilized phages up to 20 months, the use of biological saline was found to be a good rehydration solution for recovery of phages and their viability. This study showed that the lyophilized phages can be stored at 4 °C and 37 °C in their lyophilized state and can be rehydrated using biological saline to retain 80–90% activity. Morphological analysis report showed that the phages ECP311, KPP235 and ELP140 were belonging to *Phieco32likevirus*, *Podoviridae* and *Myoviridae* respectively. Post-lyophilization, the activity of the phages was retained, eventually; there was no change in morphology (data not shown) which confirmed that the excipients used for lyophilization were not detrimental. This concluded that sucrose and gelatin can be used as excipient during two-step lyophilization process to retain the phage activity during long-term storage at 4 °C and 37 °C.

### Viability of lyophilized phage cocktails

Phage cocktails can be used to cure infections caused by multiple bacteria. Therefore, preparation of phage cocktails is considered as one of the important pharmacological aspects of phage therapy. Phage cocktails are found to show promising activity in animal models^30,31^. Earlier *in vitro* studies showed that all the three phages in cocktail have promising activity against multiple bacterial infections^32,33^. This is one of the few studies to report the preparation of lyophilized cocktail of phages and to study their viability in long-term storage. The three phages (ECP311, KPP235 and ELP140) were mixed together at 1:1:1 ratio and lyophilized using 0.5 M/1.0 M sucrose, 1%/2% gelatin and 0.5 M sucrose plus 1% gelatin as excipients which showed <2 log reduction in phage cocktail activity after lyophilization (Figs 6, S1). After long-term storage, the lyophilized phage cocktail was rehydrated using biological saline that showed stable activity up to 10 months but a gradual decrease in phage viability was observed after 10 months of storage at 4 °C and 37 °C. Importantly, preparation of lyophilized phage cocktails involved the same lyophilization conditions, excipients and rehydration solution used for lyophilization of phages alone that showed promising outcomes and found to be sufficient for storage up to 5–8 months. In comparison with lyophilized phage cocktails, individual phages showed improved viability through long-term storage. At this point, it could be suggested that the therapeutic phage cocktails can be prepared by mixing individual phage powders rather than preparing lyophilized phage cocktails. In future, studies focusing on phage cocktail lyophilization should use different lyophilization conditions to retain the viability of phage cocktails, which needs further investigation.

## Conclusion

Phage therapy is one of the promising therapeutic alternatives to combat the problem of antibiotic resistance. Developing phage formulations with improved stability under physical and chemical stresses, and for long-term storage will broaden the therapeutic application of bacteriophages. This study proved that lyophilization with suitable excipients improves the viability of bacteriophages for long-term storage. We found that sucrose, gelatin and sucrose plus gelatin are beneficial for phage lyophilization. The combination of 0.5 M sucrose and 1% gelatin could maintain the phage viability with <1 log reduction in phage activity during the lyophilization process. A lyophilized cocktail preparation of the same phages using 0.5 M sucrose and 1% gelatin as excipient was found to also maintain the viability of phage cocktail up to 10 months, and further studies are needed to standardize the lyophilization conditions for preparation of phage cocktails. The studies on bacteriophage lyophilization are important for the development of novel phage formulations, for example, dry powders for inhalation and also for storage of phages at non-refrigerated conditions, during transport. More research on bacteriophage lyophilization could move phage therapy a step forward as an alternative to antibiotics.

## Materials and Methods

### Chemicals

Glucose, Sucrose, Mannitol, Gelatin, Sorbitol, Sodium chloride, Tris hydrochloride and MgSO_4_.7H_2_O were purchased from HiMedia Laboratories (chemicals), India. Polyethylene glycol MW6000 (PEG 6000) was supplied by Sigma-Aldrich chemicals. All the chemicals chosen as an excipient for phage lyophilization were previously been proven to maintain the protein viability. All the other chemicals and solvents were obtained at analytic grade from HiMedia Laboratories, India.

### Bacterial strains and bacteriophages

The bacterial strains and bacteriophages used for the study were collected from Antibiotic Resistance and Phage Therapy Laboratory, VIT, Vellore, India. The bacterial strains used for this study were *E*. *coli* ec311, *K*. *pneumoniae* kp235 and *E*. *cloacae* el140^32,33^. In total, three bacteriophages used in this study were *Escherichia* phage- ECP311, *Klebsiella* phage- KPP235 and *Enterobacter* phage- ELP140^33,34^.

### Phage preparation and plaque assay

The host bacterial strains were grown in Luria-Bertani (LB) broth (HiMedia Laboratories, India) medium for three hours at 37 °C and were used as a host for phage propagation. To the 10 mL of bacterial culture, 200 µL of phage lysate was added and incubated at 37 °C for 16 hrs. The mixture was centrifuged at 6,000 × g for 15 min and the supernatant was filtered through 0.22-micron pore sized syringe filters. The obtained phage filtrate was assayed. Briefly, to the 400 µL bacterial culture, 200 µL of phage filtrate was added and the mixture was incubated for 15 min. To the mixture, 3 mL of molten soft agar (0.75% agar) was added and poured onto a prepared LB agar plate (1.5% agar). The plates were incubated at 37 °C for 12 h and the appearance of plaques indicated the presence of phages. The plaque assay was performed for each dilution of bacteriophages, numbers of plaques were counted and PFU (Plaque Forming Units) was determined^34^. In the case of phage purification, the plaque plates were flooded with SM buffer (5.8 g NaCl; 50 mL 1 M Tris-HCl [pH 7.5]; 2 g MgSO4.7H2O; 5 mL for 1000 mL) and incubated for 4 hrs without shaking at room temperature. The buffer was collected from the plate and centrifuged at 6,000 × g for 15 min and filtered through a 0.22-micron filter^34^. The filtrate was tested using a plaque assay (as explained earlier). The phages in the filtrate were precipitated using 10% Poly-(ethylene) glycol (PEG 8000) and 1% NaCl, and allowed to precipitate at 4 °C overnight and centrifuged at 13,000 × g for 30 min. The pellet was resuspended in SM buffer or biological saline (0.85%). To evaluate the stability of bacteriophages in suspension, the purified phages in biological saline were stored at 4 °C and 37 °C and their activity was assayed for every 5 months up to 20 months^2^. All the experiments were repeated in two independent experiments.

### Lyophilization

For preparation of bacteriophages for lyophilization, six excipients and their combinations were used as follows; glucose (0.5 M and 1.0 M), sucrose (0.5 M and 1.0 M), Mannitol (0.5 M and 1.0 M), Gelatin (1% and 2%), PEG 6000 (0.1 M and 0.5 M), sorbitol (0.5 M and 1.0 M), 1.0 M glucose plus 1% gelatin and 0.5 M sucrose plus 1% gelatin. The concentrations of the excipients were based on the preliminary studies. For phage cocktail preparation, all the phage solution was mixed in an equal ratio to obtain 10^8^ PFU/mL. Two milliliters of phage solution (5.0 × 10^8^ PFU/mL) was prepared using 1500 µL of excipient and 500 µL of phage stock solution in a 10 mL vials (lyophilization bottles with stoppers) and lyophilized using a Benchtop K freeze dryer (VirTis, USA). The lyophilization cycles were used as described by Merabishvili *et al*., with small modifications^2^. Briefly, the sample holding shelves were cooled to 5 °C and the samples were precooled at −20 °C. After the samples were loaded, vials containing shelves were cooled to −30 °C (1 °C/min) and maintained for 90 min. The complete solidification of the phage solution was ensured within 90 min at −30 °C. Primary drying was maintained at −30 °C for 12 h at 100 millitorr. During secondary drying, the temperature was increased from −30 °C to 25 °C (0.1 °C/min) for 10 h at 100 millitorr. After lyophilization, the vials were tightly sealed and stored at 4 °C^2,3^. For each sample, six replicates were prepared in this study and all the experiments were repeated in two independent experiments. The standard mean for each individual experiment was compared.

### Phage stability studies

The lyophilized phage was stored at 4 °C and at 37 °C up to 20 months to determine the phage stability and the purified phage suspension was used as a control. The number of viable phages was determined using double agar overlay method and a number of plaques represent viable phages, and the lyophilized powder was assayed using 10 mg/mL that was serially diluted to ten different concentrations^14^. Phage cocktail was prepared using all the three phages at an equal concentration (10^8^ PFU/mL) and tested before and after lyophilization. The stability of phages was tested at 5 months intervals and the lyophilized phage particles were resuspended in biological saline (rehydrating solution) and the lytic activity was tested using plaque assay^14^. The initial active phage concentration (PFU/mL) and final active phage concentration (after lyophilization) were calculated using plaque assay to find out the loss in phage activity during lyophilization.

### Morphological characterization

Phage morphology was determined using Transmission Electron Microscopic (TEM) analysis^34^. The purified phage particles at 10^8^ PFU/mL were negatively stained using phosphotungstic acid [PTA; 2%; pH 7.0). Briefly, the copper grid was loaded with 10 µL of phage lysate and the liquid was allowed to absorb for 10–15 min. The remaining liquid was removed using filter paper and the prepared 2% PTA solution (staining solution) was added. The staining solution was allowed to absorb for 5 min and the excess stain was removed and the grid was washed twice with sterile water. The stained phage particles in the copper grid were allowed to dry at room temperature for 20–30 min and visualized under Transmission Electron Microscopy (FEI-TECNAI G2-20 TWIN, VIT, Vellore).

### Statistical analysis

The experiments performed to evaluate the stability of lyophilized bacteriophages were repeated in two independent experiments. The standard mean for each individual experiment was compared and the results were represented as bar graphs.

## Supplementary information

Dataset 1
